# Unveiling Skin Manifestations: Exploring Cutaneous Signs of Malnutrition in Eating Disorders

**DOI:** 10.7759/cureus.44759

**Published:** 2023-09-06

**Authors:** Mohit Wani, Swarupa Chakole, Suyash Agrawal, Anannya Gupta, Jay Chavada, Aniket G Pathade, Seema Yelne

**Affiliations:** 1 Medicine, Jawaharlal Nehru Medical College, Datta Meghe Institute of Higher Education and Research, Wardha, IND; 2 Internal Medicine, Jawaharlal Nehru Medical College, Datta Meghe Institute of Higher Education and Research, Wardha, IND; 3 Medical Student, Jawaharlal Nehru Medical College, Datta Meghe Institute of Higher Education and Research, Wardha, IND; 4 Research and Development, Jawaharlal Nehru Medical College, Datta Meghe Institute of Higher Education and Research, Wardha, IND; 5 Nursing, Shalinitai Meghe College of Nursing, Datta Meghe Institute of Higher Education and Research, Wardha, IND

**Keywords:** multidisciplinary approach, psychological aspects, treatment, diagnosis, skin health, cutaneous manifestations, malnutrition, eating disorders

## Abstract

The intricate interplay between eating disorders, malnutrition, and their cutaneous manifestations is the focal point of this comprehensive review. The review delves into the clinical significance of recognising and understanding these visible signs in the context of eating disorders. It highlights the vital role of nutrition in maintaining healthy skin and addresses the challenges associated with relying solely on cutaneous signs for diagnosis. Emphasising a multidisciplinary approach involving dermatologists, psychiatrists, and nutritionists, the review underscores the holistic nature of the treatment. Addressing psychological aspects alongside nutritional rehabilitation is underscored with a forward-looking perspective on future research avenues. This review is valuable for healthcare professionals by synthesising existing knowledge and identifying research gaps. It aims to improve the diagnosis, treatment, and preventative strategies for individuals dealing with the complex challenges of eating disorders and malnutrition.

## Introduction and background

Malnutrition, a critical consequence of eating disorders, significantly threatens physical and mental well-being. In pursuing an idealised body image, individuals afflicted by eating disorders such as anorexia nervosa, bulimia nervosa, and binge-eating disorder often engage in restrictive eating, excessive exercise, and other harmful behaviours that disrupt their nutritional intake. This disturbance in nutritional equilibrium leads to myriad health complications, some of which are outwardly evident through various cutaneous manifestations. This review aims to shed light on the intricate relationship between eating disorders, malnutrition, and the skin, highlighting the importance of recognising and understanding cutaneous signs as valuable diagnostic and therapeutic tools [1-4].

Eating disorders encompass a range of psychological conditions characterised by abnormal eating patterns and distorted perceptions of body shape and weight. Anorexia nervosa, characterised by extreme calorie restriction and fear of weight gain, often results in severe malnutrition. Bulimia nervosa involves cycles of binge eating followed by purging behaviours, which also disrupt the body's nutritional balance. Binge-eating disorder involves recurrent episodes of overeating, often leading to obesity and related health issues [5,6].

The impact of eating disorders on nutrition and health cannot be overstated. Prolonged malnutrition can lead to physiological and psychological complications, including muscle wasting, weakened bones, hormonal imbalances, cardiovascular issues, and cognitive disturbances. The skin, the body's largest organ and a direct reflection of overall health, often reveals the systemic effects of malnutrition through various dermatological manifestations [7,8].

The skin is a dynamic canvas that portrays the body's internal health status. Cutaneous signs often provide visible cues of underlying malnutrition and metabolic disturbances in individuals with eating disorders. These signs can be early indicators, prompting timely intervention and treatment before more severe health consequences arise. By identifying and understanding these skin manifestations, healthcare professionals, including dermatologists, nutritionists, and mental health specialists, can collaborate to provide comprehensive care that addresses eating disorders' nutritional and psychological aspects [9,10].

This review explores the connection between eating disorders, malnutrition, and the skin's cutaneous manifestations. By examining the specific dermatological signs associated with malnutrition, we intend to enhance awareness among medical practitioners and foster a deeper understanding of the significance of these signs in diagnosing and managing eating disorders. Additionally, we will discuss the challenges and limitations of relying solely on cutaneous signs for diagnosis and highlight the need for a holistic approach that integrates medical, psychological, and nutritional interventions.

## Review

Eating disorders and malnutrition

Definition and Classification of Eating Disorders

Eating disorders encompass a range of complex mental health conditions characterised by disturbed eating behaviours, distorted body image, and intense preoccupations with weight and shape. Three significant classifications are commonly recognised.

**Figure 1:**
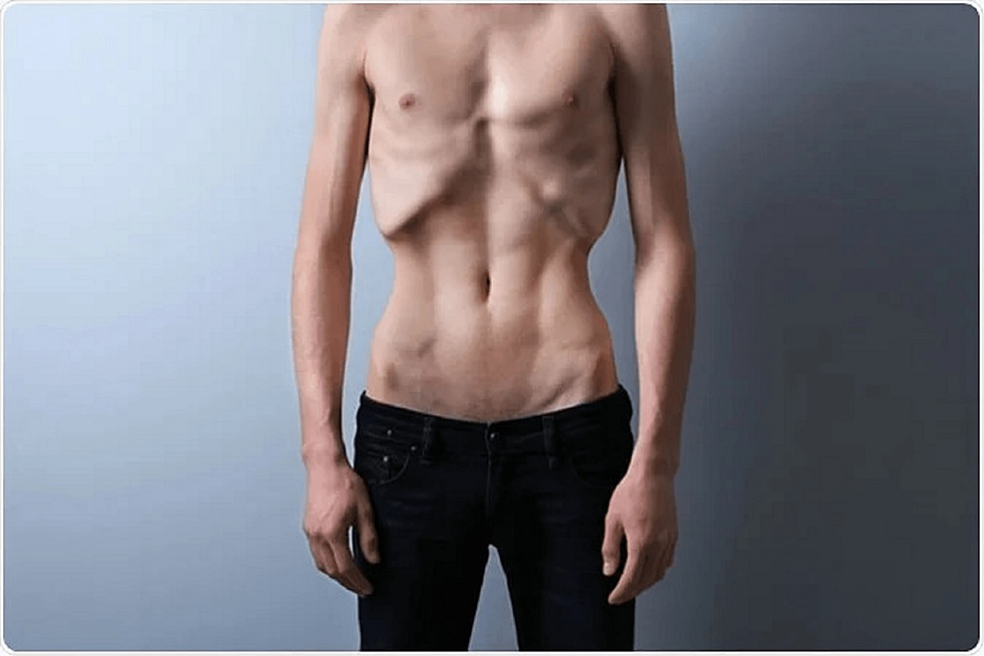
Male with anorexia Image Credit: Africa Studio/Shutterstock.

Anorexia nervosa: Anorexia nervosa is a severe eating disorder characterised by a relentless pursuit of thinness (Figure 1), often at the cost of one's health. Individuals with anorexia exhibit an extreme fear of gaining weight and a distorted body image, leading to restrictive eating habits that result in severe caloric restriction. The lifetime prevalence rates of anorexia nervosa might be up to 4% among females and 0.3% among males [11]. This deprivation of nutrients and energy gradually leads to emaciation, with visible weight loss and skeletal appearance. The malnutrition associated with anorexia nervosa affects physical health and contributes to various cutaneous signs, highlighting the critical role of nutrition in maintaining healthy skin. These signs include dry and flaky skin, brittle nails, and lanugo hair, which underscore the need for early detection and comprehensive treatment strategies [11].

**Figure 2:**
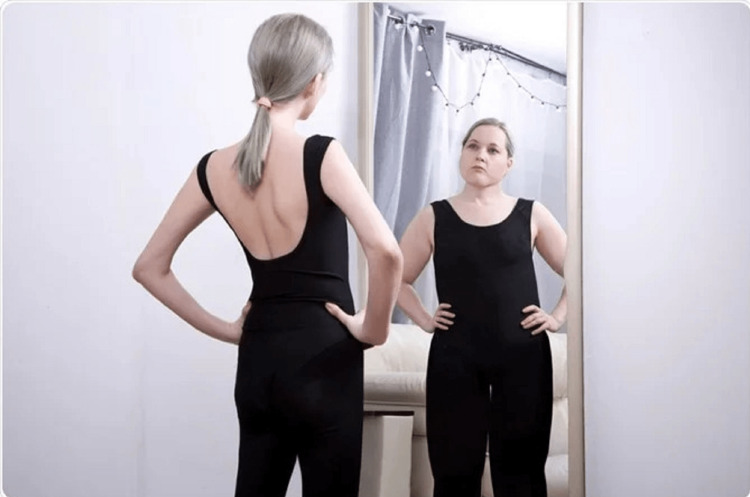
Bulimia nervosa Image Credit: Tatyana Dzemileva/Shutterstock.com.

Bulimia nervosa: Bulimia nervosa presents a complex pattern of disordered eating characterised by recurrent episodes of uncontrollable binge eating, followed by compensatory behaviours to rid the body of excess calories (Figure 2). The overall prevalence of bulimia nervosa was 0.3%. The prevalence of bulimia nervosa was five times higher among females (0.5%) than among males (0.1%) [12]. These behaviours often include self-induced vomiting, excessive exercise, or the misuse of laxatives. The cycle of bingeing and purging places significant stress on the body, disrupting its nutritional balance and leading to various health complications. Skin manifestations of bulimia nervosa may include a pale and sallow complexion due to nutrient deficiencies and potential dehydration leading to dry skin. Repeated purging behaviours can also contribute to electrolyte imbalances, impacting skin health and overall well-being [12].

**Figure 3:**
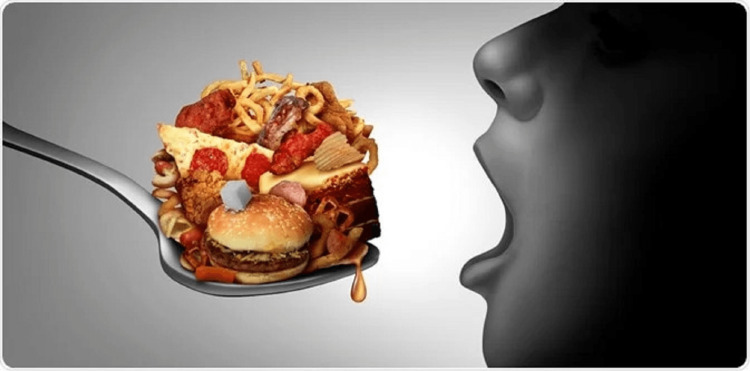
Binge-eating disorder Image Credit: Lightspring/Shutterstock.com.

Binge-eating disorder: Binge-eating disorder is characterised by recurrent episodes of consuming large amounts of food within a short span, accompanied by feelings of guilt, shame, or distress. American adults are estimated at 1.2%, compared to 0.3% for bulimia and 0.6% for anorexia [13]. Unlike bulimia nervosa, individuals with binge-eating disorders do not engage in regular purging behaviours. The excessive consumption of calories during binges can lead to weight gain and obesity, which are associated with health concerns (Figure 3). Skin manifestations in binge-eating disorder may arise due to the impact of obesity on the skin's elasticity and the potential for chafing, emphasising the interplay between nutritional health and skin integrity. Understanding these distinct eating disorder subtypes and their respective effects on skin health is essential for providing tailored interventions that address recovery's nutritional and psychological aspects [13].

**Figure 4:**
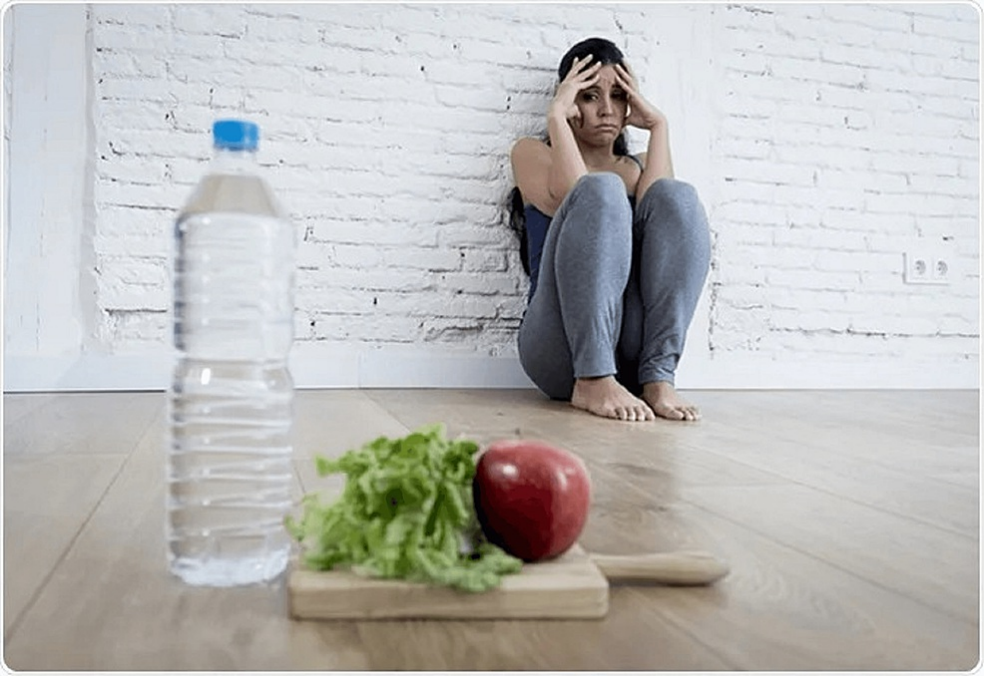
Avoidant/restrictive food intake disorder Image Credit: Marcos Mesa Sam Wordley/Shutterstock.

Avoidant/restrictive food intake disorder: Avoidant/restrictive food intake disorder (ARFID) is characterised by a limited range of accepted foods, avoidance of certain textures or smells, and/or an overall lack of interest in eating (Figure 4). Unlike other eating disorders, weight and body image concerns might not be central. Individuals with ARFID may experience nutritional deficiencies and difficulties in social situations involving food. ARFID prevalence estimates range from 0.3% to 15.5% [12].

Pica: Pica is characterised by the persistent consumption of non-nutritive, non-food substances, such as dirt, clay, or paper, over at least one month. These substances include paper, hair, cloth, chalk, and more. Pica can lead to serious health issues due to ingesting harmful or indigestible materials. The prevalence of symptoms of Pica was found to be 30.7%, accounting for 118 adolescents [13].

Overview of Prevalence and Demographic Factors

Gender: Historically, the perception that eating disorders primarily affected females prevailed, leading to a significant underrepresentation of males in clinical discussions. However, recent research has debunked this stereotype, revealing that males are also susceptible to eating disorders. While conditions like anorexia nervosa and bulimia nervosa continue to exhibit higher prevalence in females, males, too, experience these disorders, albeit at a lower frequency. Binge-eating disorder, characterised by recurring episodes of overeating, demonstrates a more balanced gender distribution, reflecting the evolving understanding that eating disorders transcend gender boundaries [14].

Age: The emergence of eating disorders spans a broad spectrum of life stages, with adolescence and young adulthood being the most commonly affected periods. The pressure to conform to societal ideals of beauty and the desire for peer acceptance during these formative years can contribute to the onset of disordered eating behaviours. However, it is essential to recognise that eating disorders are not limited to the young population. Cases have been documented in children, who may experience unique challenges due to their developmental stage, as well as in adults, underscoring the ongoing need for awareness and appropriate interventions across all age groups [15].

Socioeconomic factors: Socioeconomic status can significantly impact an individual's vulnerability to eating disorders. Cultural and societal norms, often amplified by media portrayal of unrealistic body images, can pressure specific populations to achieve an "ideal" appearance. Socioeconomic disparities can exacerbate this pressure, where limited resource access may hinder proper nutrition and contribute to body dissatisfaction. Additionally, individuals from higher socioeconomic backgrounds might face stressors related to achievement and success, which could also influence the development of eating disorders. Understanding the interplay of these factors is crucial for tailoring effective prevention and intervention strategies that consider the diverse contexts in which eating disorders arise [16].

Physiological and Psychological Factors Contributing to Malnutrition

Caloric restriction: In individuals with anorexia nervosa, caloric restriction is a defining characteristic. This extreme limitation of food intake, driven by a fear of weight gain and distorted body image, significantly reduces energy consumption. As a result, the body's access to essential nutrients becomes severely compromised. While the primary focus might be on calorie reduction, this approach inadvertently leads to an inadequate intake of vital vitamins, minerals, proteins, and other essential components required for the body's physiological functions. This deprivation sets the stage for malnutrition, resulting in various health complications, including the emergence of visible cutaneous signs [17].

Binge-purge cycles: Bulimia nervosa is marked by cycles of binge eating, where large quantities of food are consumed quickly, followed by compensatory behaviours like vomiting, laxative use, or excessive exercise to counteract caloric intake. These binge-purge cycles disrupt the body's nutrient absorption and utilisation processes, creating imbalances in electrolytes, vitamins, and minerals due to repeated purging. Nutrient absorption mechanisms in the digestive system are overwhelmed by excess intake and subsequent purging, leading to inadequate assimilation of essential elements. Over time, this pattern contributes to nutritional deficiencies, impacting overall health and manifesting as cutaneous signs of malnutrition [12].

Nutritional quality: Even in the case of binge-eating disorder, where excessive food consumption is the predominant behaviour, the nutritional quality of the consumed foods is crucial. Individuals engaging in binge eating often opt for calorie-dense, low-nutrient foods that provide momentary comfort but lack essential vitamins, minerals, and proteins. This dietary pattern produces a paradoxical scenario with abundant calories, but a nutrient deficiency is required for optimal functioning. Consequently, the body's nutritional needs are unmet, leading to malnutrition-related complications, including skin manifestations [18].

Psychological distress: The psychological component is pivotal in driving and exacerbating disordered eating behaviours. Body dissatisfaction, low self-esteem, anxiety, and depression often underlie eating disorders. These emotional stressors can perpetuate the cycle of restricted eating or binge-purge behaviours. The heightened psychological distress triggers physiological responses that impact metabolism, hormonal regulation, and digestion. These responses can contribute to disruptions in the absorption and utilisation of nutrients. The psychological distress can also lead to neglect of self-care, including adequate nutrition, further compounding the malnutrition-associated skin manifestations [19].

Metabolic adaptations: In response to prolonged malnutrition, the body initiates a series of metabolic adaptations to conserve energy and prioritise essential functions. These adaptations include slowing metabolic rate, reducing energy expenditure, and altering nutrient utilisation. While these adaptations are survival mechanisms, they contribute to a further decline in nutrient availability for bodily functions. Consequently, these adaptations can perpetuate nutritional deficiencies and hinder overall health, including the skin's vitality and integrity [20].

Skin and nutritional health

Explanation of the Role of Nutrition in Maintaining Healthy Skin

The skin serves as a mirror reflecting an individual's overall health and well-being. It is a complex organ that relies on various nutrients to function optimally. Proper nutrition is pivotal in maintaining the skin's integrity, appearance, and function. Essential nutrients are essential for cell turnover, collagen synthesis, wound healing, and protection against oxidative stress. Adequate intake of these nutrients is vital to ensure that the skin remains healthy, vibrant, and capable of performing its protective and regenerative functions [21].

Relationship Between Malnutrition and Various Skin Manifestations

**Figure 5:**
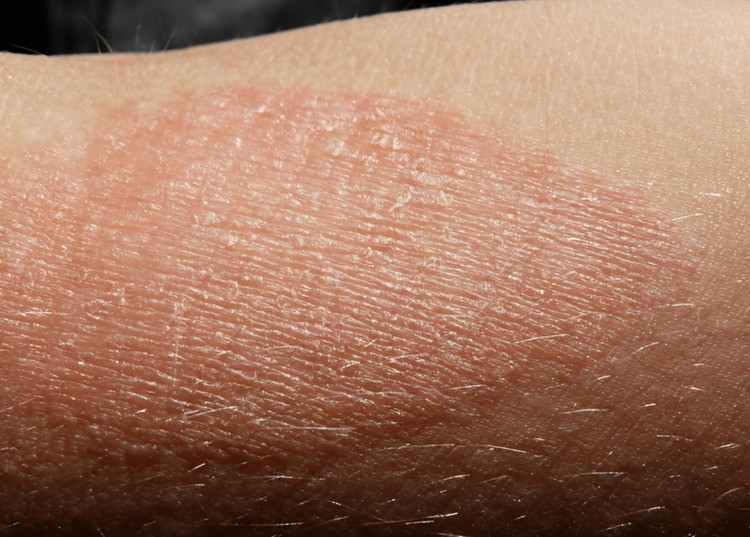
Dry and irritated skin Image credit: G.steph.rocket, 2015.

Malnutrition disrupts the delicate balance of nutrient availability, leading to a cascade of events that manifest on the skin's surface. Various skin manifestations can be observed in individuals with eating disorders and malnutrition:

Dry and flaky skin: Insufficient intake of essential fatty acids, crucial components for maintaining the skin's lipid barrier, coupled with inadequate hydration, can result in the appearance of dry and flaky skin (Figure 5). The skin's lipid barrier is a protective shield, preventing excessive water loss and guarding against external irritants. Malnutrition disrupts this barrier, leading to compromised skin barrier function. Dryness and flakiness often accompany itchiness and discomfort, signalling an underlying deficiency of key nutrients essential for maintaining the skin's moisture and integrity [22].

Brittle nails and hair: Deficiencies in vital minerals such as zinc and biotin can manifest as brittle nails and hair. Zinc plays a pivotal role in maintaining the structural integrity of nails and hair by supporting the proteins that contribute to their strength. A lack of zinc can lead to weakened nails prone to splitting and breaking and hair that becomes dry, dull, and easily breakable. Biotin, another essential nutrient, produces keratin, a protein constituent of nails and hair. Insufficient biotin levels can hinder the development of strong and healthy nails and hair [23].

Lanugo hair: Fine, downy hair may develop on the body due to chronic malnutrition. The body's adaptive mechanism aims to conserve body heat due to low body fat levels. This response is reminiscent of how certain mammals grow thicker fur in colder conditions. Lanugo hair is an insulating layer attempting to offset the body's lack of subcutaneous fat. This phenomenon is particularly prevalent in severe cases of malnutrition and often indicates a body struggling to maintain adequate energy reserves [23].

Pale and sallow complexion: A reduced intake of crucial nutrients like iron and B vitamins can result in anaemia, which manifests as a pale and sallow complexion. Iron is essential for producing haemoglobin, a protein transporting oxygen throughout the body. When iron levels are insufficient, the skin's oxygen supply diminishes, leading to a lack of colour and vitality in the complexion. Similarly, B vitamins are vital for maintaining healthy skin tone, and their deficiency can contribute to the development of a pallid and sallow appearance [24].

**Figure 6:**
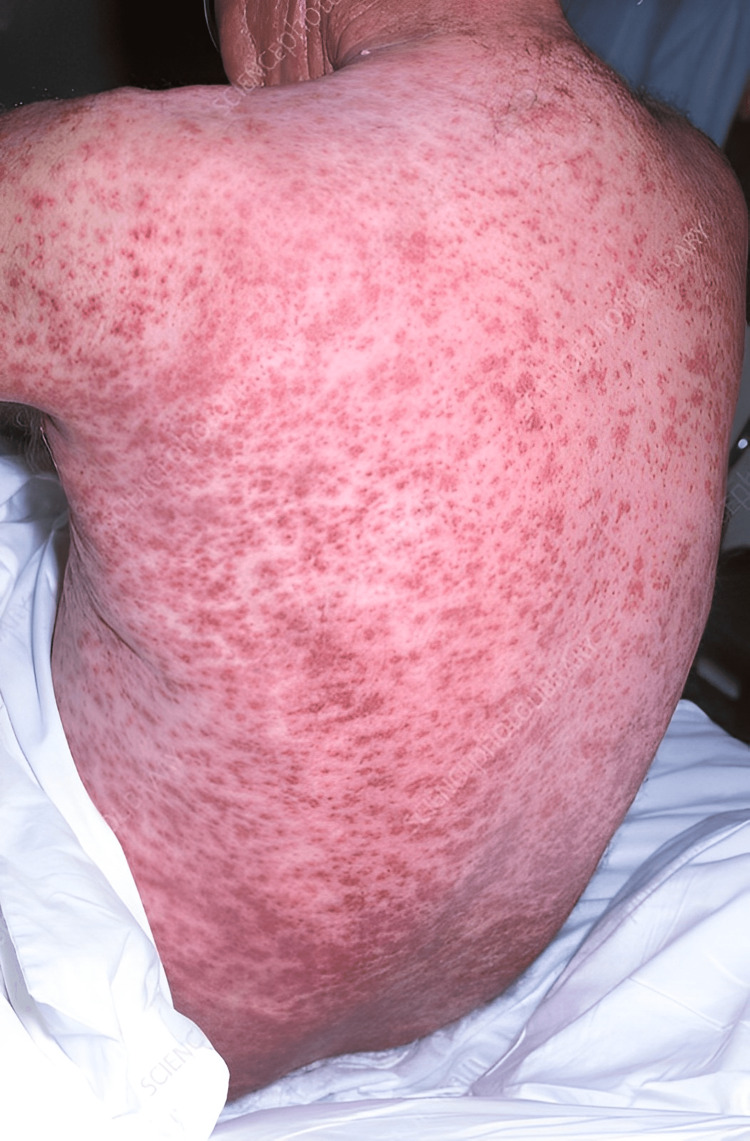
Purpura rash Credit: Dr M.A. Ansary/Science Photo Library.

Petechiae and purpura: Vitamin C, a vital antioxidant essential for collagen synthesis, is integral to maintaining the integrity of blood vessels. A deficiency in vitamin C weakens the walls of blood vessels, making them prone to rupture. This can result in the appearance of petechiae, small red or purple spots caused by tiny haemorrhages beneath the skin's surface (Figure 6). In more severe cases, purpura can develop larger areas of purple discolouration due to larger-scale bleeding. These manifestations highlight the profound impact of malnutrition on the body's physiological processes [25].

Oedema: Protein malnutrition disrupts the balance of fluids in the body, leading to oedema or swelling. Proteins in the blood are essential for maintaining the osmotic balance that prevents the excessive accumulation of fluid in the interstitial spaces. Without sufficient proteins, fluid accumulates, particularly in the extremities. Oedema can cause discomfort and impede normal bodily functions, serving as a visual and tactile representation of the body's struggle to maintain equilibrium in the face of nutritional deficiency [26].

Overview of Key Nutrients Important for Skin Health

Vitamin A: Vitamin A is pivotal in maintaining skin health through its involvement in various processes. It supports skin cell growth and repair by promoting epithelial cell differentiation, helping to replace old and damaged cells with new ones. Additionally, vitamin A regulates sebum production, which is crucial for maintaining healthy moisturisation and preventing excessive dryness or oiliness. Vitamin A contributes to a balanced, clear, radiant complexion by supporting proper cell turnover and sebum regulation [27].

Vitamin C: Vitamin C, also known as ascorbic acid, is an essential nutrient with remarkable benefits for the skin. It is a critical player in collagen synthesis, the protein responsible for maintaining the skin's structure, firmness, and elasticity. Furthermore, vitamin C supports wound healing by aiding in the formation of connective tissue and promoting the production of new skin cells. Its potent antioxidant properties protect the skin from oxidative damage caused by environmental factors such as UV radiation and pollution, helping to maintain a youthful appearance and prevent premature ageing [28].

Vitamin E: Vitamin E is renowned for its antioxidant prowess, shielding against oxidative stress and its damaging effects on the skin. It acts as a lipid-soluble antioxidant that protects cell membranes and lipids from free radical damage. Vitamin E helps prevent cellular damage, maintain skin health, and contribute to a more resilient skin barrier by neutralising free radicals from UV radiation, pollution, and other sources. This, in turn, aids in reducing the risk of inflammation and skin sensitivities [29].

B Vitamins: Biotin, niacin (B3), and riboflavin (B2) are B vitamins that play distinct roles in supporting skin health. Biotin, often called the "beauty vitamin," is essential for metabolising fatty acids and amino acids and maintaining healthy skin cells. Niacin is involved in DNA repair and helps regulate inflammation, while riboflavin supports cellular energy production and antioxidant defence mechanisms. B vitamins promote the renewal of skin cells, aid in maintaining barrier function, and contribute to an even skin tone [30].

Zinc: Zinc is crucial for skin integrity, wound healing, and inflammatory control. It supports collagen formation and DNA synthesis, vital for skin structure and repair. Zinc also regulates immune responses, which is essential for managing inflammation and preventing skin disorders. In wound healing, zinc facilitates cell proliferation and tissue repair, contributing to faster injury recovery and maintaining the skin's health and resilience [31].

Omega-3 fatty acids: Omega-3 fatty acids, commonly found in sources like fish oil and flaxseeds, are essential for maintaining the skin barrier's integrity. They support the formation of a healthy lipid layer, which prevents excessive moisture loss and keeps the skin hydrated and supple. Omega-3s also possess anti-inflammatory properties that help manage skin conditions characterised by inflammation, such as acne and eczema. Omega-3 fatty acids contribute to overall skin health and appearance by promoting barrier function and reducing inflammation [32].

Proteins: Proteins are life’s building blocks and are equally vital for the skin's structure and function. Collagen and elastin, the two major proteins in the skin, provide structural support and elasticity. Adequate protein intake ensures the availability of amino acids necessary for synthesising these proteins. Additionally, proteins contribute to wound healing, tissue repair, and immune function, which are integral for maintaining healthy skin and facilitating recovery from damage [33].

Minerals: Essential minerals like selenium, copper, and iron play diverse roles in skin health. Selenium is an antioxidant that protects the skin from oxidative stress and inflammation. Copper supports collagen synthesis and plays a role in wound healing by aiding in the formation of connective tissue. Iron is essential for oxygen transport in the blood, ensuring adequate oxygen supply to skin cells and promoting overall vitality. These minerals contribute to skin regeneration, resilience, and protection against environmental stressors [34].

Cutaneous signs of malnutrition in eating disorders

Malnutrition resulting from eating disorders can give rise to various distinctive cutaneous manifestations. Each manifestation provides valuable insights into individuals' nutritional status and overall health. In this section, we delve into the specific cutaneous signs commonly observed in individuals with eating disorders, offering detailed descriptions and explanations of their clinical presentation and underlying mechanisms [35].

Dry and Flaky Skin

Clinical presentation: The skin appears dry, rough, and flaky, often accompanied by itching and discomfort.

Underlying mechanisms: Insufficient intake of essential fatty acids is crucial for maintaining the skin's lipid barrier and preventing water loss. Reduced water intake can further exacerbate dehydration and dryness [36].

Brittle Nails and Hair

Clinical presentation: Nails become weak, brittle, and prone to splitting, while hair becomes dry, dull, and easily breakable.

Underlying mechanisms: Deficiencies in nutrients such as biotin, zinc, and protein compromise the structural integrity of nails and hair, hindering their growth and strength [37].

Lanugo Hair

Clinical presentation: Fine, downy hair called lanugo may appear on the face, arms, and back, particularly in response to low body fat.

Underlying mechanisms: The body attempts to conserve heat by growing this fine hair, responding to chronic malnutrition, and using an adaptive mechanism to maintain body temperature [38].

Pale and Sallow Complexion

Clinical presentation: The skin takes on a pale, yellowish hue, and individuals may appear visibly fatigued.

Underlying mechanisms: Reduced intake of iron and B vitamins leads to anaemia, causing inadequate oxygen transport and a pallid complexion [39].

Petechiae and Purpura

Clinical presentation: Small, red, or purple spots (petechiae) or larger areas of purple discolouration (purpura) may appear on the skin.

Underlying mechanisms: Vitamin C deficiency weakens blood vessels, making them more prone to rupture and causing bleeding into the skin [40].

Oedema

Clinical presentation: Swelling, particularly in the lower extremities, due to fluid retention.

Underlying mechanisms: Protein malnutrition disrupts the balance of fluids in the body, leading to fluid accumulation and oedema [41].

Others

In addition to the prominent cutaneous manifestations discussed earlier, several other signs can arise due to malnutrition, highlighting the systemic impact of inadequate nutrient intake:

Glossitis and oral ulcers: Malnutrition, particularly deficiencies in vitamins and minerals such as vitamin B12, iron, and folate, can lead to inflammation of the tongue, known as glossitis. This condition is characterised by redness, swelling, and discomfort. Additionally, deficiencies can impair the body's ability to maintain oral health, leading to painful oral ulcers, discomfort, and difficulty eating [42].

Delayed wound healing: Adequate nutrition is crucial for the body's repair and regenerative processes. In malnutrition, insufficient intake of critical nutrients like protein, vitamins (such as vitamin C), and minerals can hinder the body's ability to heal wounds effectively. This results in delayed wound healing, leaving individuals vulnerable to infections and complications [43].

Easy bruising: Malnutrition, especially deficiencies in vitamin K, can disrupt the body's ability to form blood clots effectively. Vitamin K is essential for proper blood clotting mechanisms, and its absence can lead to easy bruising even from minor traumas or pressure. The compromised clotting factors make blood vessels more susceptible to damage and bleeding [44].

Pruritus and dermatitis: The skin's barrier function relies on proper nutrition to maintain its integrity. Malnutrition can compromise this barrier, leaving the skin more susceptible to irritants and allergens. This vulnerability can result in pruritus (itching) and dermatitis (skin inflammation), making the skin more reactive and prone to discomfort and skin-related issues [32].

Diagnostic and clinical considerations

Diagnostic Value of Cutaneous Signs in Identifying Malnutrition

Cutaneous signs offer valuable diagnostic cues for identifying malnutrition associated with eating disorders. As visible indicators of underlying physiological disturbances, they can serve as critical early warning signs, prompting healthcare professionals to investigate further and initiate appropriate interventions. These signs not only aid in diagnosing malnutrition but also underscore the urgent need for a comprehensive assessment of the individual's nutritional and psychological status [10].

Challenges and Limitations in Using Skin Manifestations as Diagnostic Criteria

While the cutaneous signs observed in individuals with eating disorders offer valuable insights into their nutritional status and overall health, relying solely on these signs for diagnosis presents certain limitations that must be carefully considered:

Variability: Cutaneous signs can display a wide range of severity, appearance, and progression variability. This variability makes it challenging to establish uniform and consistent diagnostic criteria. What may be a pronounced manifestation in one individual might be subtle or absent in another, complicating establishing a standardised diagnostic framework [45].

Overlap with other conditions: Some skin manifestations associated with malnutrition in eating disorders can share similarities with those seen in other dermatological conditions. This resemblance can result in potential misdiagnosis if healthcare professionals do not carefully differentiate between manifestations of malnutrition and those of other unrelated conditions. Distinguishing between these overlapping presentations requires a nuanced understanding of dermatology and nutrition [46].

Delayed presentation: Not all individuals with eating disorders will exhibit noticeable cutaneous signs, particularly in the early stages of malnutrition. Cutaneous manifestations might only become apparent after a prolonged period of malnutrition, rendering them less effective as early diagnostic indicators. This delayed presentation underscores the importance of a comprehensive assessment considering multiple factors beyond skin appearance [10].

Individual differences: Genetic predisposition, overall health status, and environmental factors can influence each individual's response to malnutrition and subsequent skin reactions. Genetic variations can lead to differences in how the skin responds to nutrient deficiencies, resulting in variations in the appearance and severity of cutaneous signs among individuals [47].

Coexisting factors: The appearance of cutaneous signs can also be influenced by other concurrent health conditions an individual may have or medications they are taking. These coexisting factors can modify how malnutrition-related manifestations present on the skin, further complicating the diagnostic process and necessitating a thorough medical history assessment [48].

Importance of a Multidisciplinary Approach

Given the intricate and multifaceted nature of addressing cutaneous signs of malnutrition in eating disorders, a multidisciplinary approach is imperative for achieving accurate diagnoses and effective treatment outcomes. Within this collaborative framework, specific roles are assumed by various healthcare professionals, each contributing their expertise to the holistic care of individuals:

Dermatologists: As experts in skin health, dermatologists are pivotal in this approach. Their specialised knowledge allows them to recognise and interpret the intricate cutaneous signs associated with malnutrition. Through their expertise, they facilitate early detection, enabling timely intervention and preventing the progression of manifestations [49].

Psychiatrists/psychologists: Mental health professionals, including psychiatrists and psychologists, bring a profound understanding of the psychological underpinnings of eating disorders. They evaluate the emotional and cognitive aspects contributing to disordered eating behaviours, fostering a comprehensive understanding of the individual's condition. Their therapeutic interventions help address the root causes, promoting holistic healing [50].

Nutritionists/dietitians: The involvement of nutritionists and dietitians is pivotal in addressing the core of malnutrition. Armed with a deep understanding of nutritional science, they design personalised dietary plans tailored to the individual's needs. Focusing on nutritional rehabilitation restores optimal health rectifies nutrient imbalances and nourishes the body to support overall well-being [51].

Medical practitioners: General physicians play a vital role as orchestrators of this multidisciplinary approach. They coordinate the efforts of the various specialists, ensuring that both physical and psychological aspects are considered. Their holistic assessment considers the broader health context, integrating insights from dermatologists, mental health professionals, and nutritionists to craft a comprehensive treatment strategy [52].

Management and treatment

Overview of Treatment Strategies for Eating Disorders and Malnutrition

Treating eating disorders and associated malnutrition requires a comprehensive and holistic approach that addresses the condition's physical and psychological aspects. Treatment strategies typically include:

Medical stabilisation: At the onset of treatment, the immediate focus is resolving acute medical complications from malnutrition. These complications can range from electrolyte imbalances to cardiac irregularities. By addressing these urgent issues, healthcare professionals aim to stabilise the individual's physiological state, ensuring that they are fit for the subsequent stages of treatment [53].

Nutritional rehabilitation: Nutritional rehabilitation is a pivotal phase in the treatment process. Under the expert guidance of a dietitian, a carefully structured plan for gradual refeeding is implemented. This approach is crucial to rectify the nutritional imbalances that have resulted from the eating disorder. By providing the body with the necessary nutrients in a controlled manner, a balanced nutritional status is gradually reestablished, and a healthy weight is restored [54].

Psychological support: Acknowledging the intricate interplay of psychological factors in eating disorders, psychological support is an integral aspect of treatment. Various therapeutic modalities, such as psychotherapy and cognitive-behavioural therapy, address the underlying psychological triggers and patterns contributing to developing and maintaining the eating disorder. This facet of treatment aims to equip individuals with coping mechanisms, empower them to develop a healthier relationship with food and their bodies and foster lasting behavioural changes [50].

Medication: In cases where coexisting mental health conditions, such as depression, anxiety, or obsessive-compulsive disorder, are identified, medication may be prescribed. Integrating pharmacological interventions as part of the treatment plan can assist in managing the symptoms of these conditions, which may exacerbate or be intertwined with the eating disorder. When used with psychological therapies, medications contribute to a comprehensive approach that addresses recovery's physical and emotional aspects [55].

Family-based therapy: Family-based therapy takes a holistic approach by involving family members in the treatment process, which is particularly influential in adolescents and younger individuals. This therapeutic method acknowledges the family's role as a support system and focuses on mobilising their involvement to aid the individual's recovery journey. Family-based therapy creates a conducive environment for sustained recovery and behavioural change within the familial context by fostering open communication, understanding, and collaboration [56].

Role of Nutritional Rehabilitation in Improving Skin Health

Nutritional rehabilitation is a cornerstone of treatment, playing a crucial role in restoring the nutrients necessary for optimal skin health. Adequate intake of vitamins, minerals, proteins, and essential fatty acids supports the skin's regenerative processes, collagen synthesis, and overall barrier function. Cutaneous manifestations like dry skin, brittle nails, and hair can gradually resolve as nutritional status improves. The skin's appearance often becomes healthier and more vibrant as a reflection of improved internal health [57].

Importance of Addressing the Psychological Aspects

While addressing malnutrition is crucial, eating disorders are complex conditions driven by psychological factors. Treating the psychological aspects is essential for achieving lasting recovery. Individuals with eating disorders often have distorted body image, low self-esteem, and a fear of weight gain. Psychotherapy helps individuals explore these issues, develop healthy coping mechanisms, and foster a positive relationship with their bodies and food. By addressing the underlying psychological triggers, individuals are more likely to sustain a healthy relationship with food and maintain their physical health in the long term [58].

Future directions and research implications

Identification of Gaps in Current Knowledge and Research

Quantification and grading: Developing standardised methods to quantify and grade the severity of cutaneous manifestations entails creating a consistent framework that healthcare professionals can use to objectively assess the extent of skin changes. The diagnostic value of these signs can be enhanced by assigning a numerical or descriptive value to each manifestation, such as dryness, brittleness, or pigmentation changes. A standardised grading system would facilitate more transparent communication among medical teams, accurately assessing malnutrition's impact on the skin.

Longitudinal studies: Long-term studies focused on tracking the progression of skin manifestations during recovery from eating disorders offer valuable insights into the dynamic relationship between malnutrition, nutritional rehabilitation, and skin health. Such studies would involve observing individuals from the initial stages of treatment through their recovery journey and documenting changes in the severity and presentation of skin manifestations. Understanding how these signs evolve can aid in gauging the effectiveness of interventions and highlight the potential reversibility of cutaneous changes.

Underlying mechanisms: Delving into the molecular and cellular mechanisms that connect malnutrition to specific skin manifestations is an avenue of research that can illuminate the intricate processes underlying these changes. Exploring how nutrient deficiencies affect collagen synthesis, cellular turnover, inflammation, and other skin-related processes can provide deeper insights into the aetiology of each manifestation. This understanding could guide the development of targeted therapies that address these mechanisms directly, potentially leading to more effective treatment approaches.

Variability and genetics: Investigating the influence of genetic factors on the variability of skin manifestations in response to malnutrition is a nuanced field of research that holds the potential to enhance diagnostic accuracy. Genetic predispositions may play a role in determining how an individual's skin responds to nutritional imbalances, resulting in distinct manifestations. By identifying genetic markers associated with specific cutaneous signs, healthcare professionals could tailor diagnostic and treatment approaches to individual patients, improving personalised care and outcomes.

Suggested Areas for Future Research and Studies

Biomarkers: Biomarkers are measurable indicators that provide insight into the physiological processes occurring within the body. In the context of malnutrition and eating disorders, researchers are exploring the identification of specific biomarkers present in the skin that correlate with nutritional imbalances. These biomarkers could include specific proteins, enzymes, or molecules that change in response to malnutrition. By discovering reliable skin-based biomarkers, healthcare professionals could have objective tools for diagnosing and monitoring malnutrition in individuals with eating disorders. These biomarkers offer a less invasive and more accurate method for assessing nutritional status than relying solely on subjective clinical observations.

Predictive value: This area of research delves into whether specific cutaneous manifestations can serve as predictive indicators of the severity of malnutrition or the likelihood of relapse in individuals undergoing recovery from eating disorders. By carefully studying patterns of skin manifestations and their correlation with the progression of malnutrition, researchers aim to identify predictive factors that could help guide treatment plans. For instance, if specific skin manifestations consistently precede a worsening nutritional state or signal a potential relapse, healthcare professionals could intervene proactively to prevent further deterioration. This predictive value could significantly impact treatment strategies and improve the long-term outcomes for individuals in recovery.

Interventions: Investigating the effectiveness of interventions is crucial for guiding evidence-based treatment approaches. In this context, researchers are studying various interventions to improve the skin manifestations associated with malnutrition and the overall recovery process for individuals with eating disorders. This might involve evaluating the impact of nutritional supplementation with specific vitamins, minerals, and nutrients on skin health. Additionally, psychological therapies could be explored with nutritional rehabilitation to understand how addressing mental health aspects can positively influence skin manifestations and recovery outcomes. The goal is to identify interventions that provide comprehensive and holistic improvements in physiological and psychological well-being.

Prevention strategies: Preventing the development of severe skin manifestations and eating disorders is a crucial research focus in this area. Prevention strategies are being explored to promote a healthy body image, provide nutrition education, and implement early interventions. By targeting risk factors and educating individuals about the importance of balanced nutrition, researchers aim to reduce the prevalence of malnutrition and its associated cutaneous signs in those vulnerable to eating disorders. Early intervention programs that identify and address potential issues in body image perception and eating behaviours can contribute to minimising the development of severe skin manifestations and the overall impact of eating disorders on health.

Implications for Improving Diagnosis, Treatment, and Prevention Strategies

Enhanced diagnosis: As research advances our comprehension of the intricate relationship between cutaneous signs and malnutrition, healthcare providers are poised to refine diagnostic accuracy. With a deeper understanding of how specific skin manifestations correlate with underlying nutritional imbalances, clinicians can use this knowledge to identify malnutrition more effectively and promptly. This expedites the diagnostic process and enables timely intervention, ultimately improving patient outcomes and prognosis.

Tailored treatment: Gaining a comprehensive insight into the mechanistic basis of distinct skin manifestations enables healthcare practitioners to develop precise treatment strategies. By understanding the molecular pathways that link malnutrition to specific cutaneous changes, medical professionals can tailor interventions that target these mechanisms directly. This personalised approach optimises treatment efficacy, ensuring that the interventions are holistic and attuned to each patient’s needs.

Integrated care: The interconnected nature of the disciplines involved in addressing eating disorders and their skin manifestations underscores the importance of an integrated care approach. Collaboration among dermatologists, psychiatrists, nutritionists, and other specialists becomes paramount. Research findings further emphasise the symbiotic relationship between these disciplines, highlighting the necessity of their collective expertise to provide well-rounded and comprehensive care. This synergy ensures that patients receive a holistic treatment regimen that addresses their condition's physiological, psychological, and nutritional dimensions.

Holistic prevention: The culmination of research insights can also profoundly impact prevention strategies. By delving into the early indicators of cutaneous signs, healthcare professionals can identify individuals at risk of developing more severe skin manifestations and eating disorders. The healthcare community can proactively design preventive interventions targeting these individuals with this knowledge. Early identification and intervention can significantly reduce the prevalence and severity of skin manifestations and eating disorders, promoting a healthier society and minimising the long-term impact on individual well-being.

## Conclusions

In conclusion, this review has delved into the intricate and consequential relationship between eating disorders, malnutrition, and the distinct cutaneous signs that arise. As we have explored the manifestations such as dry skin, brittle nails, lanugo hair, pale complexion, and others, the significance of these visible cues as diagnostic indicators and windows into internal health has become evident. The critical role of nutrition in maintaining healthy skin has been underscored, emphasising how malnutrition disrupts the skin's vitality and integrity. While these cutaneous signs offer invaluable insights, it is essential to acknowledge the limitations of relying solely on them for diagnosis. Hence, a multidisciplinary approach involving dermatologists, mental health professionals, and nutrition experts remains essential for comprehensive care. The significance of addressing the psychological components of eating disorders alongside nutritional rehabilitation has been highlighted, paving the way for effective and holistic treatment strategies. Looking forward, the promise of future research lies in refining diagnostic criteria, improving treatment efficacy, and enhancing prevention efforts. By recognising the importance of cutaneous signs, collaborating across disciplines, and staying abreast of research advancements, healthcare professionals can collectively contribute to the early detection, intervention, and improved well-being of individuals grappling with eating disorders.
